# Identification of MicroRNAs and Their Target Genes Related to the Accumulation of Anthocyanins in *Litchi chinensis* by High-Throughput Sequencing and Degradome Analysis

**DOI:** 10.3389/fpls.2016.02059

**Published:** 2017-01-10

**Authors:** Rui Liu, Biao Lai, Bing Hu, Yonghua Qin, Guibing Hu, Jietang Zhao

**Affiliations:** ^1^State Key Laboratory for Conservation and Utilization of Subtropical Agro-Bioresources, South China Agricultural UniversityGuangzhou, China; ^2^Key Laboratory of Biology and Genetic Improvement of Horticultural Crops-South China of Ministry of Agriculture, College of Horticulture, South China Agricultural UniversityGuangzhou, China

**Keywords:** *Litchi chinensis*, anthocyanin, microRNA, high-throughput sequencing, *miR156a*, *LcSPL1*, LcMYB1

## Abstract

Litchi (*Litchi chinensis* Sonn.) is an important subtropical fruit in southern China and the fruit pericarp has attractive red skin at maturity, which is provided by anthocyanins accumulation. To understand the anthocyanin biosynthesis at post-transcriptional level, we investigated the roles of microRNAs (miRNAs) during fruit coloring. In the present study, four small RNA libraries and a mixed degradome library from pericarps of ‘Feizixiao’ litchi at different developmental phases were constructed and sequenced by Solexa technology. A total of 78 conserved miRNAs belonging to 35 miRNA families and 41 novel miRNAs were identified *via* high-throughput sequencing, and 129 genes were identified as their targets by the recently developed degradome sequencing. miR156a and a novel microRNA (NEW41) were found to be differentially expressed during fruit coloring, indicating they might affect anthocyanin biosynthesis through their target genes in litchi. qRT-PCR analysis confirmed the expression changes of miR156a and the novel microRNA (NEW41) were inversely correlated with the expression profiles of their target genes *LcSPL1*/*2* and *LcCHI*, respectively, suggesting regulatory roles of these miRNAs during anthocyanin biosynthesis. The target genes of miR156a, *LcSPL1*/*2*, encode transcription factors, as evidenced by a localization in the nucleus, that might play roles in the regulation of transcription. To further explore the relationship of *LcSPL1*/*2* with the anthocyanin regulatory genes, yeast two-hybrid and BiFC analyses showed that LcSPL1 proteins could interact with LcMYB1, which is the key regulatory gene in anthocyanin biosynthesis in litchi. This study represents a comprehensive expression profiling of miRNAs in anthocyanin biosynthesis during litchi fruit maturity and confirmed that the miR156- SPLs module was conserved in anthocyanin biosynthesis in litchi.

## Introduction

Anthocyanins, synthesized by a specific branch of the flavonoid pathway, which represent a large class of secondary metabolites, have a variety of functions within plants (Winkel-Shirley, 2001). Athocyanins biosynthesis is regulated by enzyme-coding structural genes, including chalcone synthase (CHS), chalcone isomerase (CHI), flavonoid-3′-hydroxylase (F3’H), flavonoid-3′,5′-hydroxylase (F3′5′H), flavanone 3-hydroxylase (F3H), dihydroflavonol 4-reductase (DFR), anthocyanidin synthase (ANS), UDP-glucose: flavonoid 3-*O*-glucosyltransferase (UFGT), and is also orchestrated at the transcriptional level by MBW transcription factor (TF) complexes consisting of MYB, bHLH (basic helix-loop-helix) and WD40 (Koes et al., 2005; Saito et al., 2013; Xu et al., 2015). Recent researches have demonstrated that the MBW complex regulates the genes that encode enzymes specifically at the late steps of the anthocyanin pathway, and the MYB TF s have been identified to be the major determinant regulatory genes in the biosynthetic steps of anthocyanin metabolism (Gonzalez et al., 2008; Lai et al., 2014; Shen et al., 2014). *AtPAP1* (*AtMYB75*), the known anthocyanin regulator originally defined in Arabidopsis, has been shown to effectively induce anthocyanin accumulation in various plant species (Zuluaga et al., 2008; Rowan et al., 2009; Li X. et al., 2010; Qiu et al., 2014). And now the *PAP1* orthologs have been identified in many horticultural crops (Cutanda-Perez et al., 2009; Chagne et al., 2012; Schaart et al., 2013; Albert et al., 2014; Jin et al., 2016). Recently, our studies suggest that the structural gene *LcUFGT* and its regulator *LcMYB1* play major roles in anthocyanin accumulation in litchi (Lai et al., 2014, 2015). In addition to MBW complex, additional TFs and regulatory genes have also been reported to affect anthocyanin biosynthesis, such as COP1 (Maier et al., 2013), JASMONATE ZIM-domain (JAZ) genes (Qi et al., 2011), the SQUAMOSA PROMOTER BINDING PROTEIN-LIKE (SPL) gene (Gou et al., 2011), and NAC (Zhou et al., 2015). These genes show an interaction with the MBW complex forming a regulatory network that modulates the production of anthocyanins.

Mature microRNAs (miRNAs) are a group of 20–24 nt non-coding small RNAs with important roles in plant growth, development, metabolism, and responses to stress by repressing their targets at post-transcriptional level (Voinnet, 2009; Iwakawa and Tomari, 2015). In plants, miRNAs are generally processed from longer single-stranded RNA hairpin precursors by Dicer-like proteins to produce a double-stranded RNA duplex, and then incorporated into the RNA-induced silencing complex where they negatively regulate their target mRNA through imperfect sequence complementarity (Rogers and Chen, 2013; Li et al., 2016). To date, a large number of miRNAs have been identified in different plant species and deposited in miRBase^1^. Acting as powerful endogenous regulators, the identification of the miRNAs and their target mRNAs could help in better understanding of the biological roles of each miRNAs on the regulatory mechanism under various plant growth and development processes. Recently, a genome-wide 5′ RACE approach, known as degradome sequencing or PARE (parallel analysis of RNA ends), has been successfully adapted to screen miRNA targets in Arabidopsis, rice, and soybean (Addo-Quaye et al., 2008; German et al., 2008; Li Y.F. et al., 2010; Song et al., 2011).

Functional analysis of miRNAs has revealed that miRNAs regulate various aspects in plant development, including leaf morphogenesis, the differentiation and development of a flower, root development, the transition from vegetative growth to reproductive growth (Jones-Rhoades et al., 2006). For example, the target genes of miR160, miR167 and miR393 regulate auxin signaling, and the regulation of AUXIN RESPONSE FACTORs (ARFs) by miR160 focuses on many aspects of shoot and root development, while miR167 appears to be important in the regulation of flowers and fruit development (Mallory et al., 2004). Besides their roles in plant development, miRNAs are also important to cope with biotic and abiotic stresses, as well as in primary and secondary metabolism (Gou et al., 2011; Li et al., 2016). Evidences suggest that miRNAs are involved in anthocyanin biosynthesis through their target genes, while only few miRNAs have been reported to be responsible for the biosynthesis of anthocyanins (Gou et al., 2011; Xia et al., 2012). In Arabidopsis, miR828 negatively controls anthocyanin accumulation by repressing the expression of *MYB75*, *MYB90*, and *MYB113*, which are involved in anthocyanin biosynthesis (Luo et al., 2012). miR858 has been identified in Arabidopsis, apple, cotton and tomato (Xia et al., 2012; Guan et al., 2014; Jia et al., 2015). miR858 is predicted to target up to 66 MYB factors in apple (Xia et al., 2012). Small tandem target mimic-mediated blockage of miR858 (STTM858) results in anthocyanin accumulation in tomato (Jia et al., 2015). It is well documented that miR156 negatively regulates anthocyanin accumulation by targeting the TF, *SPL9* (Gou et al., 2011).

In plants, miR156 targeting a subset of SPLs is highly conserved and plays important role in plant development (Nodine and Bartel, 2010). The miR156/SPLs module also participate in the biosynthesis of phenylpropanoids, as SPL9 was reported to repress anthocyanin accumulation by destabilizing the MBW complex, and directly preventing expression of anthocyanin biosynthetic genes in Arabidopsis (Gou et al., 2011). Recently, the miR156-targeted SPL has been connected to the spatiotemporal regulation of sesquiterpene biosynthesis in Arabidopsis and patchouli (Yu et al., 2015).

Litchi (*Litchi chinensis* Sonn.) is one of the most popular members of the Sapindaceae fruit with sweet, juicy flesh. The pericarp of litchi is mostly red at maturity, which is provided by anthocyanins accumulation. Apart from the delicious taste, attractive red skin of litchi is another important aspect of the fruit quality. Recently, the gene involved in anthocyanin biosynthesis and sequestration from litchi has been reported (Wei et al., 2011; Lai et al., 2014, 2015, 2016; Li et al., 2015; Hu et al., 2016). However, the mechanism of miRNAs regulating anthocyanin biosynthesis in litchi has not been reported. In the present study, we identified miRNAs in litchi pericarps during fruit ripening by using Solexa sequencing, and then analyzed the miR156/SPL module and its regulatory roles in the anthocyanin biosynthesis in litchi.

## Materials and Methods

### Plant Materials

Litchi (*L. chinesis* Sonn.) cv. ‘Feizixiao’ (FZX) was collected from the Yongfa Fruit Farm, Haikou, China. Pericarp disks of six different developmental phases (*S*_a_ and *S*_b_, green; *S*_c_, breaker; *S*_d_, half red and half yellow; *S*_e_ and *S*_f_, red) were collected between May 4th and May 29th, 2013 at 5 days intervals. All samples were immediately frozen in liquid nitrogen and stored at -80°C until use.

*Nicotiana benthamiana* plants used for subcellular localization and bimolecular fluorescence complementation (BiFC) assays were grown in greenhouses with condition of 16 h/8 h day/night at 25°C.

### Anthocyanin Analysis

The total anthocyanin content was determined according to the method published by Wrolstad et al. (1982) with some modifications. Samples were added into 6 ml of HCl/methanol (v/v) leaching solution for extraction of anthocyanin (for 5 h at 25°C in darkness). Each extraction solution diluted with pH 1.0 and pH 4.5 buffers was measured by ultraviolet scenery at 530 nm. Anthocyanin contents were calculated using a molar absorbance coefficient of 29, 600 (cyaniding-3-glucoside). Each sample was replicated three times.

### Small RNA and Degradome Libraries Construction

Total RNAs were extracted from litchi pericarps using the TRK-1001 total RNA purification kit (LC Science, Houston, TX, USA) according to the manufacturer’s instructions. Equal volumes of RNA extracts from *S*_a_ and *S*_b_ or *S*_e_ and *S*_f_ were pooled for use as S1 or S4 for sRNA library construction and sequencing. Four libraries were denoted S1 (*S*_a_ and *S*_b_), S2 (*S*_c_), S3 (*S*_d_), S4 (*S*_e_ and *S*_f_). For sRNA library construction, the sRNA fractions with the length of 10–40 nt were purified from 15% denaturing polyacrylamide gel. The purified sRNA was ligated to a 5′ adaptor and a 3′ adaptor sequentially by T_4_ RNA ligase and then transcribed into cDNA by RT-PCR following the Illumina protocol. These libraries were used for 50 bp single end sequencing using Illumina GAIIx (San Diego, CA, USA) at the LC-BIO (Hangzhou, China).

The method used for degradome library construction was previously described by German et al. (2008) with some modifications. Poly (A)+ RNA was isolated and annealed with Biotinylated Random Primers. The annealed products containing 5′-monophosphates were ligated to a 5′ adaptor and used to generate first-strand cDNA. And then we performed the single-end sequencing (36 bp) using the 5′ adapter only on an Illumina Hiseq2500 at the LC-BIO (Hangzhou, China).

### Bioinformatic Analysis

Small RNA reads were processed following the procedures as described by Yin et al. (2008). Raw reads obtained using Illumina’s Sequencing Control Studio software version 2.8 (SCS v2.8, San Diego, CA, USA) were filtered to remove low quality reads and adapters using ACGT101-miR (LC Sciences, Houston, TX, USA). After rigorous screening, all clean reads of 18–25 nt with three or more copies in frequency were mapped to specific species precursors in miRBase 20.0 by BLAST search to identify known miRNAs and novel 3 p- and 5 p- derived miRNAs.

For the degradome sequencing data, raw reads were obtained using Illumina’s Pipeline v1.5 software to remove adaptor sequences and low quality sequencing reads. CleaveLand3.0 and the ACGT301-DGEv1.0 program were used for subsequent analysis. The degradome data were mapped to the transcriptome of pericarp during litchi maturation (Lai et al., 2015). All targets were selected and categorized as I, II, III, or IV based on the abundance of the resulting mRNA tag relative to the overall profile of degradome reads that matched the target as described by Addo-Quaye et al. (2008). In addition, *t*-plots were built to analyze the miRNA targets and RNA degradation patterns according to the distribution of signatures (and abundances) along these transcripts. To uncover the miRNA-gene regulatory network on the basis of biological process and molecular function, the gene ontology (GO) analysis of the candidate target transcripts was performed using the AgriGO toolkit (Du et al., 2010).

### miRNAs and Target Gene Expression Analysis

The miRNA expression was analyzed by qRT-PCR using a published method with some modifications (Chen et al., 2005). Specific stem loop RT primer 5′-GTCGTATCCAGTGCAGGGTCCGAGGTATTCGCACTGGATACGACGTGCTC-3′ and 5′-GTCGTATCCAGTGCAGGGTCCGAGGTATTCGCACTGGATACGACGACAGA-3′ was designed for miRNA156a and a new microRNA (NEW41), respectively. Total RNA was treated with RNase-free DNaseI (TaKaRa, Dalian, China) to remove genomic DNA. For miRNA expression analysis, RNA was reverse transcribed into cDNA using the MMLV-reverse transcriptase (Invitrogen) with miRNA specific stem loop primer. For miRNA target gene expression, 1 μg of total RNA was used for the synthesizing of cDNA, incubated with oligo (dT) primer by MMLV-reverse transcriptase (Invitrogen) following the supplier’s manual. A miRNA-specific primer and a universal primer (provided in the miScript SYBR Green PCR Kit) were used for miRNA expression analysis. And two specific primers were used to amplify each miRNA target gene. All primers were listed in Supplementary Table S1. All the qRT-PCR was carried out using the miScript SYBR Green PCR Kit (TOYOBO) and performed on the ABI 7500 Real-Time PCR Systems (Applied Biosystems, USA) according to the standard protocol. The relative expression changes were calculated using the 2^-ΔΔCt^ method (Livak and Schmittgen, 2001) with *Lcactin* (HQ615689) as internal control gene. Each reaction was carried out in triplicate with different cDNA synthesized from three biological replicates.

### Gene Cloning and Sequence Analysis

Based on the Litchi Genome Sequence Resource (unpublished) and the degradome sequencing results, specific primers (Supplementary Table S2) were designed to obtain the sequence of *LcSPL1* and *LcSPL2*. The cDNA was synthesized from the total RNA of the mature pericarp of cultivar ‘FZX’ and used as the PCR templates. The PCR products were cloned into T/A cloning vector pMDH20-T (TaKaRa, Dalian, China) and transformed into *Escherichia coli* DH5α cells, the ligated vector DNAs were sequenced by Taihe Biotechnology Institute. Multiple sequence alignment was performed using ClustalX 1.83^2^ and MEGA 5 (Tamura et al., 2011).

### Subcellular Localization Analysis

The *35S::LcSPL1-GFP* and *35S::LcSPL2-GFP* vectors were constructed and transiently expressed in the leaves of *N. benthamiana* to observe the subcellular localization of LcSPL1 and LcSPL2. Briefly, the coding sequences of *LcSPL1* and *LcSPL2* without the stop codon were amplified by PCR (Primers were listed in Supplementary Table S2) and then subcloned into the pEAQ-HT-GFP vector using *Age* I in frame with the green fluorescent protein (GFP) sequence (Sainsbury et al., 2009). Then the fusion constructs and the pEAQ-HT-GFP vector as control were transformed into *Agrobacterium tumefaciens* strain GV3101 using freeze-thaw method. *Agrobacterium* cultures containing the *35S::LcSPL1-GFP*, *35S::LcSPL2-GFP* and pEAQ-HT-GFP constructs were infiltrated into the leaves of *N. benthamiana*. Two days after infiltration, leaf protoplasts were isolated and observed with a fluorescence microscope (Zeiss Axio Observer D1). All transient expression assays were repeated at least three times.

### Transcriptional Activation Analysis in Yeast Cells

The full-length coding regions of *LcSPL1*/*2* were amplified and fused to the GAL4 DBD to generate pGBKT7-LcSPL1 and pGBKT7-LcSPL1 constructs. The fusion plasmids together with the positive control (p-53+T-antigen) and negative control (pGBKT7) were transformed into the Y2H Gold yeast strain using the PEG/LiAc method. The transformed yeast cultures were dropped onto plates of minimal medium without Trp (SD/–Trp) and SD/–Trp–His–Ade plates at 30°C for 3 days, and their growth status and the α-galactosidase activity were tested to further determine the transcriptional activation of each protein. For the protein showing autoactivation in yeast cells, the C-terminus deletion and N-terminus deletion constructs were constructed and transformed into the yeast cells for transcription activation activity as described above. The primers used for transcriptional activation analysis were listed in Supplementary Table S2.

### Yeast Two-Hybrid (Y2H) Assay

Yeast two-hybrid assays were performed to study the interaction of LcSPL1/2 with LcMYB1 or LcbHLH1/2/3 using the Matchmaker^TM^ Gold Y2H system (Clontech) according to the manufacturer’s instructions. Full length of LcSPL1 showed autoactivation in yeast cells. The C-terminus deletion construct, LcSPL1-N were created in bait vector for Y2H analysis. *LcSPL1-N* and *LcSPL2* was fused to the DNA-binding domain in the bait plasmid pGBKT7, respectively, and *LcMYB1* or *LcbHLH1/2/3* was independently cloned into activation domain in the prey vector pGADT7. Primers were shown in Supplementary Table S2. Different pairs of bait and prey constructs were then co-transformed into the yeast strain Y2HGold using the PEG/LiAc method, and the yeast cells were grown on SD medium without leucine and tryptophan (SD/-Leu/-Trp). Transformed colonies were screened for growth on quadruple dropout SD medium lacking adenine, histidine, leucine and tryptophan (SD/-Ade/-His/-Leu/-Trp), with X-α-Gal and Aureobasidin A (AbA) background. X-α-Gal was used to assay for α-galactosidase activity to confirm the positive interactions.

### BiFC Assay

The entire coding sequences of *LcSPL1*/*2* and *LcMYB1* (without their stop codons) were amplified and subcloned into the pEAQ-CYFP and pEAQ-NYFP vectors described by Lai et al. (2016). The expression of target genes alone was used as negative controls. All constructed vectors were transformed into *A. tumefaciens* strain GV3101 and transiently expressed in leaves of *N. benthamiana*. The transformed leaf protoplasts were isolated and used for YFP fluorescence observation. All the transient expression assays were repeated at least three times. The primers used in the BiFC assay were listed in Supplementary Table S2.

## Results

### High-Throughput Sequencing of sRNAs

**FIGURE 1:**
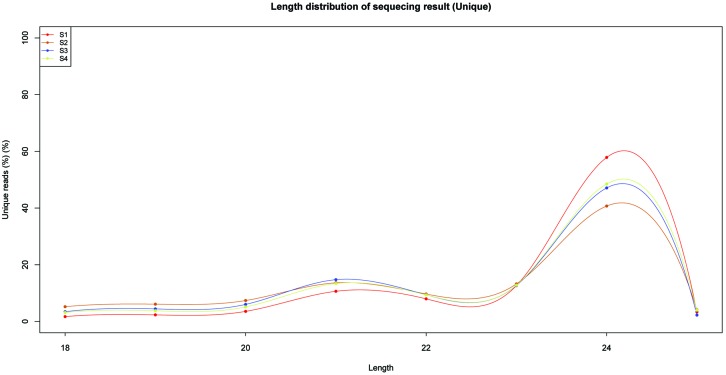
**Length and size distribution of unique sequences in four sRNA libraries in litchi**.

To identify miRNAs involved in the biosynthesis of anthocyanins in litchi, four sRNA libraries were constructed from the litchi pericarps at different developmental phases, and sequenced by the Solexa technology. After removing of low quality tags and contaminated adapter sequences, a total of 3036722 (S1), 1583580 (S2), 2802666 (S3), and 2836881 (S4) clean reads were obtained, respectively (**Table 1**). The size distribution of the sRNAs was summarized in **Figure 1**. The majority of sRNAs were 24 and 21 nt, with 24 nt having the highest abundance, and most conserved miRNAs belonging to the group of 21 nt sRNAs.

**Table 1:** Data summary of four sRNA sequencing from litchi pericarp.

	S1		S2		S3		S4	
	Total	% of Total	Total	% of Total	Total	% of Total	Total	% of Total
Raw reads	7,766,629	100.00	15,013,45	100.00	13,295,93	100.00	13,309,79	100.00
3ADT& filter	812,985	10.47	2,578,744	17.18	3,150,740	23.70	3,430,728	25.78
Junk reads	30,708	0.40	26,410	0.18	43,837	0.33	33,762	0.25
Rfam	495,921	6.39	1,955,439	13.02	1,199,645	9.02	1,224,164	9.20
mRNA	600,337	7.73	1,597,537	10.64	1,176,682	8.85	1,129,129	8.48
Repeats	11,537	0.15	32,687	0.22	46,235	0.35	23,231	0.17
valid reads	5,979,350	76.99	9,531,000	63.48	8,004,118	60.20	7,710,341	57.93
rRNA	390,298	5.03	1,655,989	11.03	953,492	7.17	829,060	6.23
tRNA	70,554	0.91	177,811	1.18	159,637	1.20	310,541	2.33
snoRNA	5,175	0.07	17,190	0.11	12,434	0.09	11,386	0.09
snRNA	4,606	0.06	8,091	0.05	8,233	0.06	9,872	0.07
other Rfam RNA	25,288	0.33	96,358	0.64	65,849	0.50	63,305	0.48

### Identification of Conserved miRNAs in Litchi Pericarp

To identify the conserved miRNAs in litchi pericarp, the clean reads were mapped onto the genome of litchi, and then all the retained sequences were compared with known miRNA precursor or mature miRNA sequences in miRBase 20.0 allowing no more than two mismatches. The unique sequences mapping to specific species mature miRNAs in hairpin arms were identified as conserved miRNAs. A total of 78 conserved miRNAs were identified in the four sRNA libraries (Supplementary Table S3). Conserved miRNAs were found to be important in plant development, growth, and many other biological processes (Jones-Rhoades et al., 2006). In the present study, 35 highly conserved miRNA families were identified. According to the sequencing results, the expression levels of the conserved miRNAs were highly variable. Among them, lch-miR156a, lch-miR159, lch-miR166a, lch-miR403a, and lch-miR396b had relatively high number of reads, whereas lch-miR169 family members expressed at a lower level (Supplementary Table S3).

### Identification of Novel miRNAs in Litchi Pericarp

The primary criterion for identification of novel miRNA was the ability of its sequence to fold-back into a stable hairpin structure. Novel miRNAs were identified by analyzing the precursors using the Mfold web server (Zuker, 2003). All the results were filtered using the strict criteria defined by Meyers et al. (2008). In total, 41 novel miRNAs were predicted (Supplementary Table S4). The 34 novel miRNAs were 18–25 nt long, and most of them were 24 nt long (**Figure 1**). Finally, 119 miRNAs, including conserved and new candidates, were identified in litchi pericarps of ‘FZX’ during fruit ripening, from the four libraries (S1/S2/S3/S4).

### Target Genes Identification for miRNAs in Litchi by Degradome Sequencing

In the present study, degradome sequencing technology was performed to identify miRNA targets for both conserved and new miRNAs (Addo-Quaye et al., 2008). Two degradome libraries (S1S4 and S2S3) were constructed and sequenced using the Solexa Analyzer. A total of 8127257 and 7112553 raw reads were yielded, respectively. After removing the low quality and repeat sequences, 3551258 and 3818329 unique reads were obtained. The remaining sequences were mapped to the litchi pericarp transcriptome (Lai et al., 2015) (Supplementary Table S5). Finally, 3524213 and 3788804 sequences could be mapped perfectly onto the reference database. All these mapped sequences were analyzed to identify candidate target gene for miRNAs.

The sliced target transcripts were categorized into five groups according to the relative abundance of the tags at the target mRNA sites. In total, 129 targets were identified for conserved and novel miRNAs (Supplementary Table S6). These targets included ARFs, NAC domain TF, GRAS family TF, MYB TF, SPLs, AP2-like factors, SCLs and MADS-box factors (Supplementary Table S6), which were reported to play important roles in the plant growth and development. For example, miR166 targeted class III homeodomain leucine zipper (HD-ZIPIII) protein has been shown to be involved in the asymmetry development of leaves in maize (Juarez et al., 2004). The TF ARF10 was targeted by miR160, and ARF10 could alter hormone sensitivity during seed germination and seedling growth (Liu et al., 2007).

### Identification of miRNAs Target Related to Anthocyanins Biosynthesis in Litchi Pericarp

**FIGURE 2:**
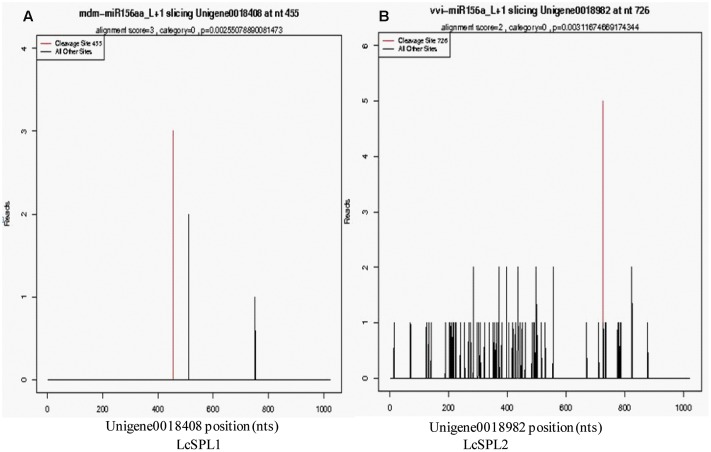
**Target plots of the targets cleaved by the miR156a.** The T-plots show the distribution of the degradome tags along the full-length of the target mRNA sequence. The red line represents the predicted cleavage site of the corresponding miRNAs. **(A)** Cleavage features in *LcSPL1* mRNA by miR156a from the degradome library. **(B)** Cleavage features in *LcSPL2* mRNA by miR156a from the degradome library.

Based on the results of miRNA identification and its target prediction (Supplementary Table S6), two miRNAs, miR156a and a novel miRNA-NEW41, were found to be related to anthocyanin biosynthesis in litchi pericarp. miR156a was found to target SPL TF (LcSPL1 and LcSPL2) (**Figure 2**), and miRNA-NEW41 was predicted to target LcCHI. SPL TF has been reported to be involved in anthocyanin biosynthesis in Arabidopsis (Gou et al., 2011), whereas LcCHI is an important structural gene in the biosynthesis of anthocyanins in litchi pericarp (Wei et al., 2011).

In S1, S2, S3, and S4 library, miR156a and miRNA-NEW41 were found to be differentially expressed (Supplementary Tables S3 and S4). Compared with the expression levels in S1 (green pericarp), the expression levels of both miR156a and miRNA-NEW41 were up-regulated and then down-regulated in the S4 (red pericarp). These results indicated that miR156a and miRNA-NEW41 might be involved in the anthocyanins biosynthesis in litchi.

### Expression Analysis of miRNAs and Their Targets by qRT-PCR

**FIGURE 3:**
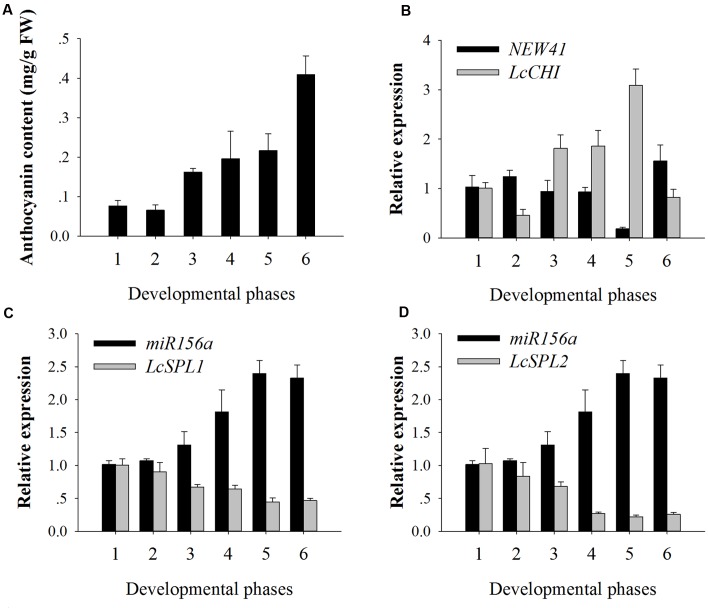
**Anthocyanin contents and relative expressions of *miRNAs* and their target genes. (A)** Anthocyanin contents of ‘FZX’ litchi during fruit maturation. **(B)** Expression analysis of miRNA-NEW41 and its target gene *LcCHI* in the pericarp of ‘FZX’ litchi. **(C)** Expression analysis of *miR156a* and its target genes *LcSPL1* in the pericarp of ‘FZX’ litchi. **(D)** Expression analysis of *miR156a* and its target genes *LcSPL2* in the pericarp of ‘FZX’ litchi. The reference gene, *Lcactin*, was used to normalize gene expression levels under identical conditions. The vertical bars represent the standard error of triplicate experiments.

To validate the results obtained by high-throughput sequencing, miR156a and miRNA-NEW41 were selected for further confirmation by qRT-PCR. The total anthocyanin content showed a trend of increasing in litchi during fruit maturation (**Figure 3A**). The expression levels of miRNA-NEW41 was slightly decreased and reached the lowest at sample *S*_e_, while its corresponding target gene *LcCHI* showed increased expression (**Figure 3B**). In parallel with the accumulation of anthocyanins, the expression patterns of miR156a were enhanced as the fruit developed toward full maturity, while its target genes *LcSPL1* and *LcSPL2* were reduced (**Figures 3C,D**). The expression patterns between miR156a and its targets (*LcSPL1* and *LcSPL2*) were complementary, which further validated the regulatory role of miR156a on its targets *LcSPL1* and *LcSPL2*. Together, the above results indicated the involvement of miR156a in the accumulation of anthocyanins in litchi, and was selected for further analysis.

### Isolation and Subcellular Localization of LcSPL1/2

In Arabidopsis, miR156 and its target *SPL9* negatively regulates anthocyanin accumulation (Gou et al., 2011). In the present study, two SPL TFs, *LcSPL1* and *LcSPL2* were isolated and characterized. The full length 915 bp *LcSPL1* (GenBank accession number KY305107) and 552 bp *LcSPL2* (accession no. KY305108) were isolated using the cDNA samples from red pericarp of ‘FZX’. The *LcSPL1* gene encoded a putative protein of 304 amino acids with a predicted molecular weight of 34.435 KDa, and the isoelectric point was 7.23. The predicted molecular weight of LcSPL2 (183 amino acids) was 22.376 kD, and the isoelectric point was 8.34. Both LcSPL1 and LcSPL2 belong to the SBP family based on the protein functional domain analysis (data not shown).

**FIGURE 4:**
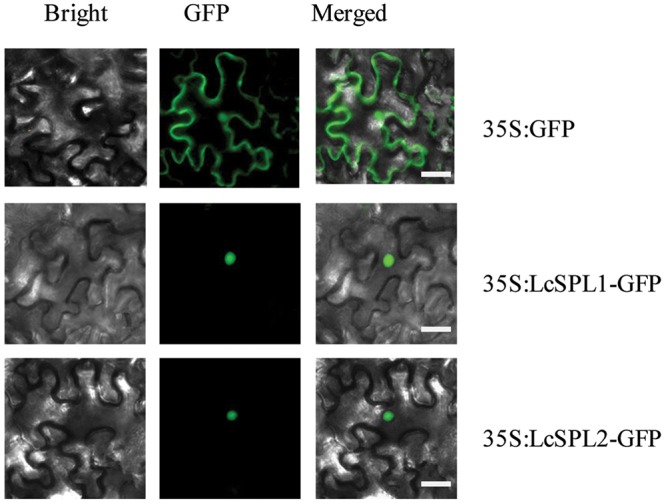
**Subcellular localization of LcSPL1 and LcSPL2 as revealed using GFP fusion proteins.** Bar = 20 μm.

SQUAMOSA PROMOTER BINDING PROTEIN-LIKEs, as TF, are expected to be localized in the nucleus. To determine the subcellular localization of LcSPL1 and LcSPL2, a 35S-LcSPL1-GFP and 35S-LcSPL1-GFP fusion protein was analyzed. Fluorescence from 35S-GFP was discerned in both cytoplasm and nucleus, whereas fluorescence from the 35S-LcSPL1-GFP and 35S-LcSPL1-GFP fusion was detected only in the nucleus, demonstrating that LcSPL1 and LcSPL2 are localized in the nucleus (**Figure 4**). The results indicated that LcSPL1 and LcSPL2 were both nuclear-localized proteins.

### Transcriptional Activation Ability of LcSPL1/2 in Yeast

**FIGURE 5:**
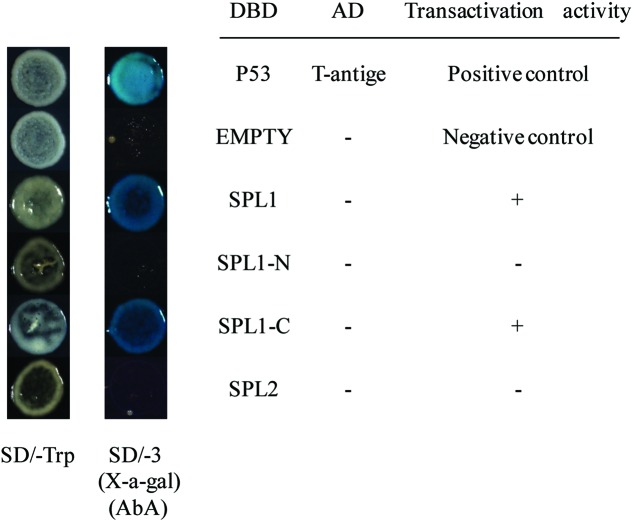
**Analysis of the transcriptional activation ability of *LcSPL1/2* in yeast cells.** The full length construct of LcSPL1/2 and the N-and C-terminal derivatives of *LcSPL1* were cloned into the pGBKT7 vector to create the DBD-LcSPL1/2, DBD-LcSPL1-N, and DBD-LcSPL1-C constructs, respectively, and then expressed in the yeast strain Y2H Gold. The plasmids pGBKT7-53+pGADT7-T and pGBKT7 were used as positive and negative controls, respectively. Yeast clones transformed with all the constructs above were grown on SD media minus Trp, Ade and His (-TAH), with X-a-Gal and Aureobasidin A (AbA) background for 3 days at 30°C.

To identify the transcriptional activation abilities of LcSPL1 and LcSPL2, a GAL4-responsive reporter system in yeast cells was performed. The yeast cells with vector pGBKT7 (empty) and cells that have been successfully transfected with the vector construct containing DBD-LcSPL2 could not grow on media minus Trp, Ade and His with Aureobasidin A background (**Figure 5**). While the yeast cells containing DBD-LcSPL1 and DBD-P53+T-antigen (positive control) grew well on the same media and showed α-galactosidase activity (**Figure 5**). These results confirmed that the DBD-LcSPL1 could act as a transcriptional activator and LcSPL2 had no transcriptional activation. To further identify the transcriptional activation domains of LcSPL1, the coding region of *LcSPL1* was separated into two fragments, which were the N-and C-terminal regions of *LcSPL1*, and fused with GAL4-BD, and then transformed into yeast strain Y2H Gold. As shown in **Figure 5**, yeast cells harboring DBD-LcSPL1-C showed α-galactosidase activity. The result indicated that the activation domain of *LcSPL1* was localized at C-terminus, and the N-terminal was used for protein interaction in Y2H.

### The Interaction of LcSPL1/2 with LcMYB1 or LcbHLH 1/2/3

**FIGURE 6:**
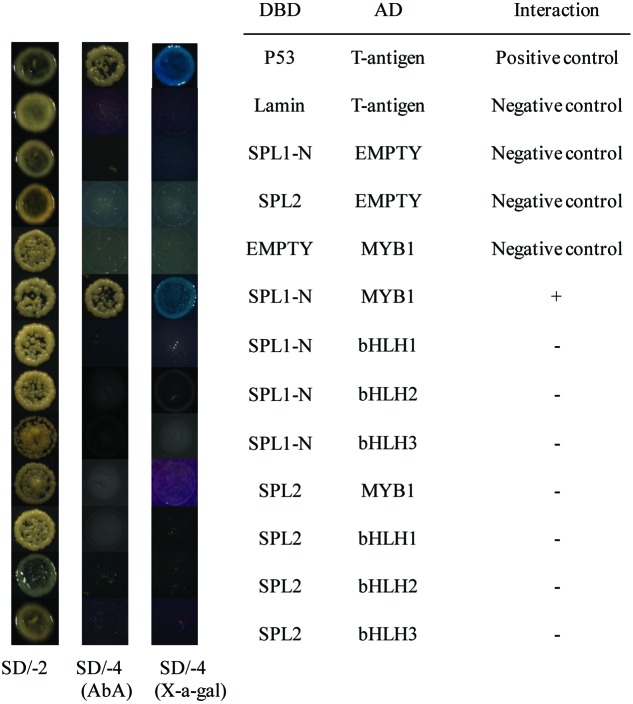
**Interaction of LcSPL1/2 with LcMYB1 or LcbHLH1/2/3 detected in Y2H assays.** The combinations of LcSPL1/2 and LcMYB1 or LcbHLH1/2/3 fused to pGBKT7 (BD) or pGADT7 (AD), respectively, was co-transformed to the Y2HGold yeast strain. All of the yeast clones were grown on appropriate media (SD/-Leu/-Trp and then SD/-Ade/-His/-Leu/-Trp) to test for activation of the reporter gene. The plasmids pGBKT7-53+pGADT7-T, pGBKT7-Lam+pGADT7-T, DBD–SPL1/2+pGADT7-T, pGBKT7+AD-MYB1 were used as positive and negative controls, respectively.

Recent report has suggested that *SPLs*, the targets of the miR156, are involved in anthocyanin biosynthesis pathways through directly preventing expression of anthocyanin biosynthetic genes by destabilizing the MBW complex (Gou et al., 2011). Based on our previously published results, *LcMYB1* might play major roles in the MBW complex determining anthocyanin accumulation in litchi (Lai et al., 2014). Recently, two LcbHLH TFs interacting with LcMYB1 in regulating late structural genes of anthocyanin biosynthesis in litchi during anthocyanin accumulation has been reported (Lai et al., 2016). In the present study, to explore interaction of LcSPL1/2 with LcMYB1 or LcbHLH1/2/3, the Y2H has been employed. The co-transformed colonies were selected on SD medium lacking leucine and tryptophan (SD/-Leu/-Trp), and screened for growth on another SD medium without adenine, histidine, leucine, and tryptophan (SD/-Ade/-His/-Leu/-Trp) supplement with X-α-Gal and Aureobasidin A (AbA) background. As shown in **Figure 6**, yeast cells co-transformed with the positive control (pGBKT7-53+pGADT7-T) and LcSPL1 with LcMYB1, could grow on selective medium (synthetic medium lacking tryptophan, leucine, histidine, and adenine) supplement with X-α-Gal and AbA background, and turned blue at the presence of X-α-Gal. In contrast, the yeasts co-transformed with the control and the yeast cells harboring LcSPL2 with LcMYB1 combinations could not (**Figure 6**). These results suggested that LcMYB1 was able to form complex with LcSPL1, but not with LcSPL2.

As shown in **Figure 6**, yeast cells co-transformed the positive control (pGBKT7-53+pGADT7-T) could grow on selective medium (synthetic medium lacking tryptophan, leucine, histidine, and adenine) supplemented with X-α-Gal and AbA background, and turned blue at the presence of X-α- Gal, but the yeasts co-transformed with the control and the yeast cells harboring LcSPL1/2 with LcbHLH1/2/3 combinations could not, indicating no direct interaction between LcSPL1/2 and LcbHLH1/2/3.

**FIGURE 7:**
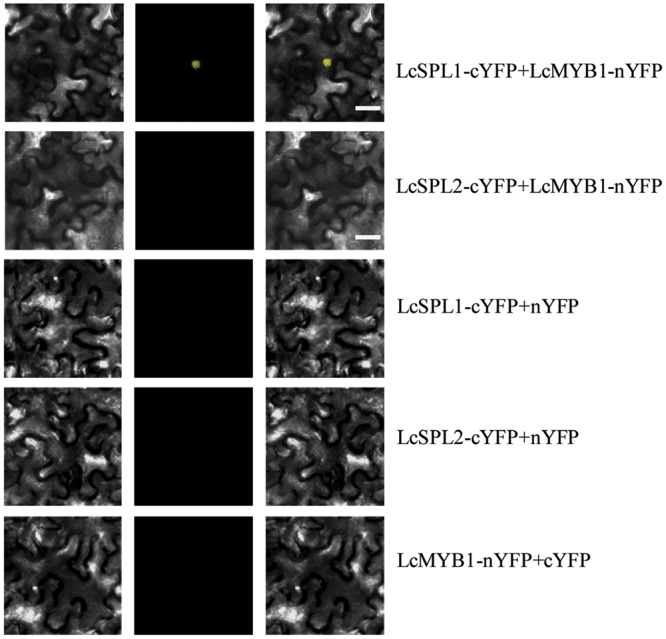
**Bimolecular fluorescence complementation assay of the LcSPL1 and LcMYB1 interaction in *Nicotiana benthamiana*.** Bright field, YFP and merged field were shown for each transformation combination. Bar = 20 μm.

Subsequently, BiFC analysis was performed to further confirm the interactions between LcSPL1/2 and LcMYB1 observed in the Y2H assays (**Figure 7**). LcSPL1/2 tagged with pSPYNE (split YFP C-terminal fragment expression) and LcMYB1 tagged with pSPYCE (split YFP N-terminal fragment expression) were transiently co-expressed in *N. benthamiana* leaves by *Agrobacterium*. As shown in **Figure 7**, strong YFP fluorescent signal was detected in the nucleus of leaf epidermal cells expressing LcSPL1-cYFP and LcMYB1-nYFP, but not in the control or LcSPL2-cYFP and LcMYB1-nYFP. The BiFC assay further demonstrated the *in vivo* interaction between LcSPL1 and LcMYB1, which was consistent with the Y2H assays.

## Discussion

### High-Throughput Sequencing

miRNAs play important roles in plant development, adaptation to biotic and abiotic stress, as well as in secondary metabolism (Jones-Rhoades et al., 2006; Gou et al., 2011; Li et al., 2016). Litchi miRNAs and targets had been identified in litchi fruit subjected to ambient storage and post-cold storage shelf life (Yao et al., 2015). However, whether miRNAs are involved in litchi anthocyanin biosynthesis is still unknown. Therefore, identification of litchi miRNAs and their target genes associated with anthocyanin biosynthesis was performed in the present study using the high-throughput deep sequencing technology. In this study, we constructed four sRNA libraries and two mixed degradome libraries from pericarps of ‘FZX’ litchi of different developmental phases. In general, 24 nt sRNAs were the most abundant, followed by 21 nt, which was consistent with the length distribution of sRNAs reported in apple and litchi (Xia et al., 2012; Yao et al., 2015). Finally, a total of 78 known miRNAs belonging to 35 highly conserved miRNA families as well as 41 new miRNA were identified in litchi (Supplementary Tables S3 and S4). Different miRNAs showed different expression levels during litchi fruit maturity, indicating miRNAs might be involved in litchi fruit development. Moreover, we identified a total of 129 potential targets of the conserved and new miRNAs by the recently developed degradome sequencing approach (Supplementary Table S6). The majority of these target genes are conserved in plant species, which had been reported to play important roles in plant development.

### miRNAs and Their Targets Related to Litchi Anthocyanins Biosynthesis

Accumulating evidences suggest that miRNAs are involved in anthocyanin biosynthesis, such as miR156 and miR858 (Gou et al., 2011; Xia et al., 2012; Jia et al., 2015). In this study, miR156a and a novel miRNA-NEW41 were found to be related to the anthocyanin biosynthesis in litchi based on the sRNA and degradome sequencing. The target gene of miR156a is *LcSPL*, a family of plant-specific TFs. And miRNA-NEW41 target *LcCHI*, which is the important structural gene of anthocyanin biosynthesis in litchi. qRT-PCR confirmed that the expression of miRNA-NEW41 was essentially complementary to that of the target *LcCHI* (**Figure 3B**). As the miRNA targeted *CHI* in the regulation of anthocyanin biosynthesis has not been reported in plant, the roles of miRNA-NEW41 in litchi anthocyanin biosynthesis are deserved to be further studied.

Recently, Gou et al. (2011) reported that SPL9 competed with TT8, a bHLH, for binding to PAP1, and could suppress *DFR* expression by interfering with a MYB-bHLH-WD40 transcriptional activation complex, and then influence the anthocyanin biosynthesis in Arabidopsis. In this investigation, we analyzed the expression profile of miR156a and its corresponding targets *LcSPL1* and *LcSPL2* in the different development phases of litchi pericarp, and the results showed that the expression level of miR156a was in parallel with the accumulation of anthocyanins, whereas its targets *LcSPL1*/*2* showed a reverse trend (**Figures 3C,D**). These results indicated that miR156a might also be related to the accumulation of anthocyanins in litchi. However, the functions of miR156a in litchi are unknown. Transgenic plants ectopically expressing the miR156a or its targets *LcSPL1*/*2* in Arabidopsis might provide an efficient platform to study the function of miRNA. For example, Sun et al. (2013) reported that transgenic Arabidopsis plants ectopically expressed *Md-miRNA156h* gene exhibited late flowering and long vegetative growth.

Previous studies showed that miR858 participated in anthocyanin biosynthesis in Arabidopsis, apple and tomato (Luo et al., 2012; Xia et al., 2012; Jia et al., 2015). However, the expression of miR858a maintained a low level during litchi fruit maturity (Supplementary Table S3). Whether miR858 is responsible for anthocyanin biosynthesis in litchi is worth for further study.

### *LcSPL* Genes Analysis

SQUAMOSA PROMOTER BINDING PROTEIN-LIKE is a family of plant-specific TFs that have been identified in various species, such as Arabidopsis (Unte et al., 2003), tomato (Salinas et al., 2012), populous (Li and Lu, 2014), and *Citrus* (Shalom et al., 2015). In plants, miR156 and its target gene *SPL* have previously been shown to play important roles in several plant developmental processes, such as flowering time and phase change, organ size, and fertility (Wang et al., 2008; Wang et al., 2009; Xing et al., 2010). Here, we described a critical role of miR156a/SPL in regulating the biosynthesis of anthocyanins in litchi. miR156a and two target genes, which were *LcSPL1* and *LcSPL2*, were obtained from the sRNA and degradome sequencing. Subcellular localization indicated that LcSPL1 and LcSPL2 were all localized in the nucleus (**Figure 4**), where they participated in the regulation of anthocyanin biosynthesis. *SPLs* responsible for anthocyanin biosynthesis have also been reported in Arabidopsis (Gou et al., 2011).

### LcSPL1 Interacting with LcMYB1 in Regulating Anthocyanin Biosynthesis

Anthocyanin biosynthesis is known to be controlled by both enzyme-coding structural genes and their regulated genes, which are influenced by hormones, light, temperature, and nutrition (Koes et al., 2005; Saito et al., 2013). The data presented here revealed a role for *LcSPL1* in regulating the expression of *LcMYB1*, thereby controlling anthocyanin biosynthesis. In the present study, Y2H and BiFC assays confirmed that LcSPL1 could physically interact with LcMYB1 (**Figures 6** and **7**), which has been proved to play major roles in anthocyanin biosynthesis in litchi (Lai et al., 2014). However, there was no interaction between LcSPL1/2 and LcbHLL1/2/3 (**Figure 6**). Recently, LcbHLH1/3 interacting with LcMYB1 in regulating late structural genes of anthocyanin biosynthesis in *Nicotiana* and *Litchi* has been reported (Lai et al., 2016). In previous study, SPL9 was reported to destabilize the MBW complex to repress anthocyanin biosynthesis (Gou et al., 2011). However, the WD40 related to anthocyanin biosynthesis in litchi has not been identified. Therefore, whether the LcSPL1/2 could bind WD40 in litchi is worth for further research. Our results, together with previous reports, clearly suggest that *LcSPL1* is involved in litchi anthocyanin biosynthesis through interaction with its regulatory gene *LcMYB1*.

Previous studies indicated that the expression of *LcUFGT*, *LcF3H*, *LcDFR*, *LcGST*, and their regulatory gene *LcMYB1* were up-regulated as the fruit developed toward full maturity (Wei et al., 2011; Lai et al., 2014; Li et al., 2015; Hu et al., 2016). In the present study, the expression of miR156a was enhanced with the accumulation of anthocyanins, while its target genes *LcSPL1*/*2* were opposite (**Figures 3C,D**). Taken together, these results suggest the up-regulation of miR156a lead to the down-regulation of *LcSPL1*, which negatively regulate anthocyanin biosynthesis via interaction with *LcMYB1*. To the best of our knowledge, this is the first report that miRNAs are involved in the anthocyanin biosynthesis in litchi, and our findings reveal a novel mechanism on regulation of anthocyanin biosynthesis in litchi. Here, we report that miR156a-targeted LcSPL1 functionally interfere with anthocyanin biosynthesis through interaction with LcMYB1.

## Author Contributions

RL, GH, and JZ designed the research. RL, BL, and BH performed the experiments. RL, YQ, GH, and JZ analyzed the data and wrote the manuscript. All of the authors read and approved the final manuscript.

## Conflict of Interest Statement

The authors declare that the research was conducted in the absence of any commercial or financial relationships that could be construed as a potential conflict of interest.

## Footnotes

1 http://www.mirbase.org/

2 http://www.ebi.ac.uk
