# Supplementary material for: Identification of MicroRNAs and Their Target Genes Related to the Accumulation of Anthocyanins in Litchi chinensis by High-Throughput Sequencing and Degradome Analysis
Source: Front Plant Sci. 2017 Jan 10;7:2059. doi: 10.3389/fpls.2016.02059 (PMC5223483; doi:10.3389/fpls.2016.02059)
Supplement: Supplementary file 1 [file Table_1.DOCX]

| Table S1 Primers of qRT-PCR for miRNAs and target genes expression | |
| --- | --- |
| miRNAs | Primers |
| miR156a | 5′-AGCGCGTTGACAGAAGAGAGG-3′ |
| NEW41 | 5′-AGCGCGGCTTTCTCTTCTTCT-3′ |
| Target genes |  |
| *LcSPL1* | 5′-TCAGGAGATTAGTTTGAGCCACAT-3′ |
|  | 5′-CTGCCATCAGAGACCAAGGG-3′ |
| *LcSPL2* | 5′-CAAAGGCTCAGGTTGTCGTC-3′ |
|  | 5′-TCGTCGTTCATTGTGTCCAG-3′ |

| Table S2 Primers for gene cloning and plasmid construction | | |
| --- | --- | --- |
| Gene cloning | | Primer |
| LcSPL1 | 5′-ATGGAATCATTAGCATCTGGTTCTT-3′ | |
|  | 5′-CTACTCCCACGAGAAGGATAGTGTC-3′ | |
| LcSPL2 | 5′-ATGGATGGCAAAGGCAAG-3′ | |
|  | 5′-TTATCTGATCTGGAAGTGTTTGAAC-3′ | |
| Subcellular localization | | |
| LcSPL1-Gfp | 5′-CAAATTCGCGACCGGTATGGAATCATTAGCATCTGG-3′ | |
|  | 5′-TGCTAGCCATACCGGTCTCCCACGAGAAGGATAGTG-3′ | |
| LcSPL2-Gfp | 5′-CAAATTCGCGACCGGTATGGATGGCAAAGGCAAGAAC-3′ | |
|  | 5′-TGCTAGCCATACCGGTTCTGATCTGGAAGTGTTTGAAC-3′ | |
| Y2H |  | |
| BD-SPL1 | 5′-CATGGAGGCCGAATTCATGGAATCATTAGCATCTGGTTCTT-3′ | |
|  | 5′-GCAGGTCGACGGATCCCTACTCCCACGAGAAGGATAGTGTC-3′ | |
| BD-SPL1N | 5′-CATGGAGGCCGAATTCATGGAATCATTAGCATCTGGT-3′ | |
|  | 5′-GCAGGTCGACGGATCCTTATTGATGATTGGGAAAGAA-3′ | |
| BD-SPL1C | 5′-CATGGAGGCCGAATTCATGGGTACCAAATTTATACCGT-3′ | |
|  | 5′-GCAGGTCGACGGATCCCTACTCCCACGAGAAGGATAGT-3′ | |
| BD-SPL2 | 5′-CATGGAGGCCGAATTCATGGATGGCAAAGGCAAG-3′ | |
|  | 5′-GCAGGTCGACGGATCCTTATCTGATCTGGAAGTGTTTGAAC-3′ | |
| AD-MYB1 | 5′-GGAGGCCAGTGAATTCATGTCGCATTTACTTGGTG-3′ | |
|  | 5′-CGAGCTCGATGGATCCTTACTTTGCATTGTCTTCTTCTATA-3′ | |
| AD-bHLH1 | 5′-GGAGGCCAGTGAATTC ATGGCTGGTGTTGTTCAAAATC-3′ | |
|  | 5′-CGAGCTCGATGGATCC TCAAAACTTACCAGCAATCTTCC-3′ | |
| AD-bHLH2 | 5′-GGAGGCCAGTGAATTC ATGGCTGGGCCGCCCAGTA-3′ | |
|  | 5′-CGAGCTCGATGGATCC TTAGGGTATTATTTGATGTATTGC-3′ | |
| AD-bHLH3 | 5′-GGAGGCCAGTGAATTC ATGGCTACTACTGGGGTTC-3′ | |
|  | 5′-CGAGCTCGATGGATCC TCAACACTTCCAAATGACTCTG-3′ | |
| BiFC |  | |
| LcSPL1-cYFP | 5′-CAAATTCGCGACCGGTATGGAATCATTAGCATCTGG-3′ | |
|  | 5′-GCACGCTGCCACCGGTCTCCCACGAGAAGGATAGTG-3′ | |
| LcSPL2-cYFP | 5′-CAAATTCGCGACCGGTATGGATGGCAAAGGCAAGAAC-3′ | |
|  | 5′-GCACGCTGCCACCGGTTCTGATCTGGAAGTGTTTGAAC-3′ | |
| MYB1-nYFP | 5′-CAAATTCGCGACCGGTATGTCGCATTTACTTGGTGC-3′ | |
|  | 5′-TGCTCACCATACCGGTCTTTGCATTGTCTTCTTC-3′ | |

|  |
| --- |

Table S3 Conserved miRNAs identified in litchi pericarp during fruit ripening

| miR_name | miR_seq | | len | | | S1(norm) | | S2(norm) | S3(norm) | S4(norm) |
| --- | --- | --- | --- | --- | --- | --- | --- | --- | --- | --- |
| vvi-miR156a | | TTGACAGAAGAGAGGGAGCAC | | 21 | 4351.41 | | 7642.97 | | 15766.96 | 14005.61 |
| vvi-miR156b | | CTGACAGAAGAGAGGGAGCAC | | 21 | 2.77 | | 2.33 | | 6.51 | 2.03 |
| mtr-miR156c | | GCTTACTCTCTATCTGTCGCC | | 21 | 1.85 | | 20.21 | | 18.22 | 2.44 |
| vvi-miR156f | | TTGACAGAAGATAGAGAGCAC | | 21 | 242.67 | | 96.40 | | 163.98 | 846.01 |
| mdm-miR156t | | TTGACAGAAGAGAGAGAGCAC | | 21 | 9.23 | | 3.11 | | 9.54 | 3.25 |
| ath-miR157a | | GCTCTCTATGCTTCTGTCATC | | 21 | 2.31 | | 11.66 | | 4.77 | 13.02 |
| aly-miR157d | | GCTCTCTACTCTTCTGTCATC | | 21 | 0.46 | | 0 | | 1.30 | 0 |
| ppe-miR159 | | TTTGGATTGAAGGGAGCTCTA | | 21 | 40443.39 | | 29223.82 | | 60886.63 | 58495.41 |
| mdm-miR159a | | CTTGGATTGAAGGGAGCTCC | | 20 | 3.23 | | 139.94 | | 0 | 1.22 |
| ath-miR159b | | TTTGGATTGAAGGGAGCTCTT | | 21 | 172.54 | | 106.51 | | 188.28 | 347.35 |
| vvi-miR159c | | TTTGGATTGAAGGGAGCTCTAA | | 22 | 41.98 | | 9.33 | | 27.76 | 28.88 |
| mdm-miR160a | | TGCCTGGCTCCCTGTATGCCA | | 21 | 100.57 | | 0 | | 16.48 | 65.89 |
| vvi-miR162 | | TCGATAAACCTCTGCATCCAG | | 21 | 2564.16 | | 1397.83 | | 2354.74 | 3846.11 |
| bna-miR162a | | TCGATAAACCTCTGCATCCAGT | | 22 | 47.52 | | 10.11 | | 8.68 | 42.71 |
| vvi-miR164a | | TGGAGAAGCAGGGCACGTGCAT | | 22 | 7.84 | | 2.33 | | 0.43 | 6.10 |
| ath-miR164b | | CATGTGCCCGTCTTCCCCATC | | 21 | 54.44 | | 188.14 | | 157.91 | 34.17 |
| vvi-miR166a | | TCTCGGACCAGGCTTCATTCC | | 21 | 45703.19 | | 26732.14 | | 3979.37 | 16041.74 |
| csi-miR166e | | GGAATGTTGTCTGGCTCGAGGA | | 22 | 99.65 | | 62.97 | | 49.45 | 113.07 |
| vvi-miR167a | | TGAAGCTGCCAGCATGATCTGT | | 22 | 5.54 | | 4.66 | | 1.74 | 12.20 |
| mdm-miR167b | | TGAAGCTGCCAGCATGATCT | | 20 | 29.30 | | 76.58 | | 25.81 | 58.98 |
| cpa-miR167c | | TGAAGCTGCCAGCATGATCTTA | | 22 | 71.74 | | 72.69 | | 87.85 | 170.02 |
| aly-miR167d | | AGGTCATCTTGCAGCTTCAAT | | 21 | 17.07 | | 4.66 | | 14.32 | 11.39 |
| ptc-miR167f | | AGATCATGTGGCAGTTTCACC | | 21 | 6.92 | | 9.33 | | 5.21 | 10.98 |
| vvi-miR168 | | TCGCTTGGTGCAGGTCGGGAA | | 21 | 6787.32 | | 412.82 | | 1525.72 | 10532.88 |
| ath-miR168a | | CCCGCCTTGCATCAACTGAAT | | 21 | 3160.22 | | 363.06 | | 571.77 | 3262.44 |
| ath-miR169a | | GGCAAGTTGTCTTTGGCTAC | | 20 | 1.38 | | 21.77 | | 24.29 | 0 |
| vvi-miR169b | TGAGCCAAGAATGGCTTGCCGGCC | | | 24 | 22.61 | | 3.89 | | 0.87 | 13.42 |
| mdm-miR169c | | TAGCCAGGGATGACTTGCCT | | 20 | 0 | | 45.09 | | 0.87 | 2.44 |
| vvi-miR169f | | CAGCCAAGGATGACTTGCCGA | | 21 | 0 | | 1.55 | | 0 | 0 |
| cme-miR169r | | AGCCAAGAATGACTTGCCGGC | | 21 | 0 | | 0 | | 0.87 | 1.63 |
| ath-miR170 | | TATTGGCCTGGTTCACTCAGA | | 21 | 27.22 | | 17.10 | | 17.79 | 45.55 |
| cpa-miR171a | | TGATTGAGCCGTGCCAATATC | | 21 | 78.43 | | 254.22 | | 279.81 | 72.94 |
| mdm-miR171i | | TGAGCCGAACCAATATCACTC | | 21 | 0.92 | | 57.53 | | 1.74 | 2.03 |
| gma-miR171k | | TTGAGCCGCGCCAATATCACT | | 21 | 32.29 | | 29.54 | | 22.56 | 6.24 |
| ppe-miR172a | | GTAGCATCATCAAGATTCACA | | 21 | 9.23 | | 9.33 | | 44.25 | 29.69 |
| vvi-miR172d | | AGAATCTTGATGATGCTGCAT | | 21 | 554.54 | | 115.84 | | 981.29 | 1160.02 |
| vvi-miR172c | | GGAATCTTGATGATGCTGCAG | | 21 | 0.92 | | 0 | | 0 | 2.03 |
| rco-miR319a | | TTGGACTGAAGGGAGCTCCT | | 20 | 86.73 | | 13.99 | | 38.18 | 91.92 |
| vvi-miR319b | | TTGGACTGAAGGGAGCTCCC | | 20 | 388.45 | | 146.16 | | 76.35 | 402.67 |
| ath-miR319c | | CTTGGACTGAAGGGAGCTCCTT | | 22 | 1.85 | | 1.55 | | 0.87 | 3.66 |
| vvi-miR319g | | ATTGGACTGAAGGGAGCTCC | | 20 | 11.99 | | 1.55 | | 0.87 | 10.17 |
| vvi-miR390 | | AAGCTCAGGAGGGATAGCGCC | | 21 | 210.37 | | 171.81 | | 99.34 | 109.01 |
| ath-miR390b | | CGCTATCCATCCTGAGTTTCC | | 21 | 13.84 | | 22.55 | | 12.15 | 5.69 |
| ath-miR390a | | CGCTATCTATCCTGAGTTTCA | | 21 | 3.23 | | 7.00 | | 2.60 | 10.17 |
| vvi-miR393a | | TCCAAAGGGATCGCATTGATC | | 21 | 55.36 | | 50.53 | | 66.81 | 78.09 |
| ath-miR393b | | ATCATGCTATCTCTTTGGATT | | 21 | 10.61 | | 41.20 | | 104.98 | 23.59 |
| vvi-miR394a | | TTTGGCATTCTGTCCACCTCC | | 21 | 19.38 | | 10.11 | | 4.77 | 20.74 |
| vvi-miR395a | | CTGAAGTGTTTGGGGGAACTC | | 21 | 555.92 | | 64.53 | | 1909.21 | 2261.06 |
| aly-miR395e | | GTTCCCCGGAGCACTTCATTG | | 21 | 19.84 | | 16.33 | | 66.81 | 35.39 |
| mdm-MIR395d | | GTTCCCTCGACCACTTCATTG | | 21 | 4.15 | | 5.44 | | 4.77 | 5.29 |
| vvi-miR396a | | TTTCCACAGCTTTCTTGAACAA | | 22 | 0 | | 4.66 | | 0.87 | 0 |
| vvi-miR396b | | TTCCACAGCTTTCTTGAACTT | | 21 | 4690.04 | | 2375.07 | | 8179.56 | 4170.28 |
| lus-miR396d | | CCCACAGCTTTATTGAACT | | 19 | 0 | | 0 | | 1.74 | 0 |
| vvi-miR397a | | TCATTGAGTGCAGCGTTGATG | | 21 | 7.84 | | 31.10 | | 17.35 | 19.12 |
| vvi-miR398a | | TTGTGTTCTCAGGTCACCCCT | | 21 | 6.92 | | 10.11 | | 8.68 | 2.44 |
| vvi-miR398b | | TGTGTTCTCAGGTCGCCCCTG | | 21 | 42.44 | | 38.87 | | 140.56 | 55.32 |
| vvi-miR399a | | TGCCAAAGGAGAATTGCCCTG | | 21 | 24.91 | | 7.00 | | 22.56 | 66.30 |
| vvi-miR399b | | TGCCAAAGGAGAGTTGCCCTG | | 21 | 76.12 | | 16.33 | | 63.34 | 212.72 |
| ptc-miR399c | | TTGCCAAAGGAGATTTGCTCA | | 21 | 0 | | 4.66 | | 1.30 | 0.41 |
| vvi-miR399e | | TGCCAAAGGAGATTTGCCCGG | | 21 | 1.85 | | 0 | | 0.87 | 16.68 |
| vvi-miR399i | | CGCCAAAGGAGAGTTGCCCTG | | 21 | 95.96 | | 32.65 | | 245.97 | 539.33 |
| vvi-miR403a | | TTAGATTCACGCACAAACTCG | | 21 | 17204.96 | | 8039.47 | | 11961.11 | 23321.54 |
| vvi-miR408 | | ATGCACTGCCTCTTCCCTGGC | | 21 | 94.11 | | 98.73 | | 284.58 | 80.13 |
| vvi-miR477a | | ACCTCCCTCAAAGGCTTCCAATG | | 23 | 3.69 | | 13.99 | | 26.90 | 4.88 |
| vvi-miR477b | | ACTCTTTCTCAAGGGCTTCC | | 20 | 0 | | 0 | | 3.47 | 0.81 |
| gma-miR482b | | TCTTCCCTACACCTCCCATACC | | 22 | 16.61 | | 9.33 | | 10.41 | 21.15 |
| ppe-miR482f | | TCTTCCCTACTCCACCCATGCC | | 22 | 7127.79 | | 16780.18 | | 14748.80 | 3443.85 |
| cme-miR530a | | TGCATTTCCACCTGCACCTTA | | 21 | 0.92 | | 0 | | 0.43 | 0.41 |
| csi-miR530b | | CTGCATTTGCACCTGCATCTG | | 21 | 1.85 | | 22.55 | | 20.82 | 5.29 |
| vvi-miR535a | | TGACAACGAGAGAGAGCACGC | | 21 | 1369.27 | | 328.86 | | 2713.50 | 3305.56 |
| ppe-miR827 | | TTAGATGACCATCAACAAACG | | 21 | 363.54 | | 203.69 | | 273.74 | 899.30 |
| vvi-miR828a | | TCTTGCTCAAATGAGTATTCCA | | 22 | 3.69 | | 0.78 | | 13.45 | 9.76 |
| vvi-miR3630 | | TGGGAATCTCTCTGATGCAT | | 20 | 4.15 | | 52.87 | | 5.21 | 10.58 |
| gma-miR482 | | TCTTTCCTACACCTCCCATACC | | 22 | 11.99 | | 9.33 | | 13.88 | 14.64 |
| ptc-miR1448_ | | TTCTTTCCAACACCTCCCATACC | | 23 | 653.27 | | 1365.18 | | 1890.56 | 910.28 |
| stu-miR3627 | | TCGCAGGAGAGATGGCACCTAG | | 22 | 0.46 | | 0 | | 1.30 | 3.66 |
| csi-miR3954 | | TGGACAGAAAAATCACGGTC | | 20 | 0.92 | | 0 | | 1.30 | 2.03 |

Table S4 Novel miRNAs identified in litchi pericarp during fruit ripening

| miR_name | miR_seq | len | CG% | dG | S1(norm) | S2(norm) | S3(norm) | S4(norm) |
| --- | --- | --- | --- | --- | --- | --- | --- | --- |
| NEW1 | TGTGGGAGGATTGGACAGAGC | 21 | 45.80 | -65.60 | 818.43 | 756.45 | 655.06 | 957.05 |
| NEW2 | TGGGTGAAAGATGCAGCAAAA | 21 | 36.40 | -60.80 | 1916.43 | 547.32 | 2137.40 | 2161.81 |
| NEW3 | TGGCTGTATCTTTCATCCACT | 21 | 36.40 | -60.80 | 36771.54 | 16388.35 | 34206.21 | 50820.27 |
| NEW4 | TTTTGATCAAATGTCCAAGGCT | 22 | 34.40 | -63.70 | 2643.97 | 2040.77 | 4261.35 | 3385.28 |
| NEW5 | TTTTATTGGATGGACGTACGGATG | 24 | 32.60 | -105.30 | 1209.65 | 507.67 | 580.88 | 560.89 |
| NEW6 | TCTTCCCTACTCCACCCATGCCT | 23 | 42.00 | -88 | 291.11 | 164.82 | 502.79 | 94.36 |
| NEW7 | TATTAGAGTTTTCGTCATCTTT | 22 | 40.70 | -114.10 | 104.73 | 349.85 | 632.50 | 140.32 |
| NEW8 | TTGTCAGAGTTGTGATTCTCT | 21 | 34.10 | -94.10 | 306.33 | 866.06 | 839.00 | 654.85 |
| NEW9 | GTCTGTCGGAACCTGTTGAGATCG | 24 | 29.40 | -88.80 | 511.17 | 105.73 | 98.91 | 414.06 |
| NEW10 | GTTAAGTGGTTGTGCACCTCTCCT | 24 | 34.20 | -74 | 617.28 | 363.84 | 570.03 | 458.80 |
| NEW11 | AGCAAAACATGGGGACGGATC | 21 | 38.00 | -50.50 | 1027.88 | 352.96 | 282.85 | 897.26 |
| NEW12 | TCTGTCCCTTTGTTTTGTTGC | 21 | 38.00 | -50.50 | 255.59 | 1507.45 | 1036.81 | 335.56 |
| NEW13 | ACTGGTGGACTTGGTACTCGAACC | 24 | 38.50 | -70.60 | 23550.77 | 4307.00 | 4852.64 | 24980.21 |
| NEW14 | TCTAGAACAACGAGTTAATGAATT | 24 | 27.50 | -117.30 | 2297.96 | 3119.85 | 2213.75 | 1433.34 |
| NEW15 | CGGAATTAACTGAGATAGGACTCT | 24 | 36.10 | -124.80 | 1735.58 | 365.39 | 1125.31 | 831.37 |
| NEW16 | TCGGATCGTCTTCCTCTCTGT | 21 | 51.10 | -54.30 | 8177.81 | 1049.54 | 1137.46 | 7603.56 |
| NEW17 | AGAGAGAAAGGCGATCTGATT | 21 | 51.10 | -54.30 | 592.37 | 209.91 | 718.40 | 994.88 |
| NEW18 | ATTTAGAACAATGAGTTAATGAAT | 24 | 29.10 | -124 | 1192.58 | 1013.00 | 964.37 | 743.52 |
| NEW19 | CACGAGCTATCCATTCTATGAA | 22 | 37.30 | -267.10 | 916.23 | 486.67 | 361.80 | 388.43 |
| NEW20 | TGCGTCAGGTAATACATGGAC | 21 | 44.20 | -62 | 3637.71 | 369.28 | 1265.00 | 898.48 |
| NEW21 | CAAATACAGTAGGCTCTCTTCT | 22 | 35.20 | -162.10 | 1186.58 | 789.88 | 1940.45 | 1096.97 |
| NEW22 | CTAGGTACTGTCCCCGGGCCC | 21 | 54.40 | -177.30 | 120.41 | 403.49 | 502.36 | 126.50 |
| NEW23 | TACCGTCCTAGTCTCAACCATA | 22 | 54.90 | -23.80 | 1652.54 | 8414.97 | 2991.58 | 3580.92 |
| NEW24 | ACCAGGGATCGGCGGATGTTGC | 22 | 54.90 | -23.80 | 1787.25 | 10809.47 | 4066.57 | 5521.06 |
| NEW25 | GCTTTCTCTTCTTCTGTCAGC | 21 | 46.90 | -50.40 | 63.20 | 1698.70 | 1151.78 | 152.12 |
| NEW26 | ATTCCCACTGTCCCTGTC | 18 | 47.10 | -25.20 | 16.61 | 662.38 | 267.23 | 94.36 |
| NEW27 | GGAGGCAGCGGTTCATCGATC | 21 | 44.90 | -40.30 | 123.64 | 18.66 | 44.25 | 130.97 |
| NEW28 | TCTCGGACCAGGCTTAATTCC | 21 | 48.20 | -11.80 | 151.32 | 27.99 | 16.05 | 56.94 |
| NEW29 | TTCCCCTGAACACTTCATTGG | 21 | 45.50 | -55.30 | 14.76 | 41.98 | 113.23 | 37.42 |
| NEW30 | AGCTGCTGAGCTATGGATCCC | 21 | 46.00 | -67.20 | 324.79 | 256.55 | 234.26 | 296.92 |
| NEW31 | TGATTGAGCCGTGCCAATATC | 21 | 37.20 | -46.80 | 78.43 | 254.22 | 279.81 | 72.94 |
| NEW32 | TCATGCGATCCCTTAGGAATT | 21 | 40.70 | -68.10 | 33.22 | 46.65 | 154.00 | 58.16 |
| NEW33 | TCCTCAAAGGCTTCCAGTATTC | 22 | 35.00 | -53.60 | 436.89 | 243.34 | 228.19 | 365.66 |
| NEW34 | CCTGGGATTGGCTTTGGGCTT | 21 | 55.50 | -79.90 | 20.30 | 101.07 | 71.15 | 104.12 |
| NEW35 | ATTCTAACCTTGTGTCAGGACC | 22 | 55.50 | -79.90 | 40.60 | 413.60 | 242.94 | 257.06 |
| NEW36 | CGTTGTAGTATAGTGGTGAGTA | 22 | 48.00 | -40.70 | 1.38 | 16.33 | 9.98 | 204.18 |
| NEW37 | GTTGTAGTATAGTGGTAAGTATT | 23 | 43.30 | -34 | 11.07 | 28.77 | 18.65 | 148.87 |
| NEW38 | GTTGTAGTATAGTGGTGAGT | 20 | 44.10 | -44.20 | 18.92 | 46.65 | 39.04 | 274.55 |
| NEW39 | GTTGTAGTATAGTGGTGAGTATT | 23 | 42.90 | -47.20 | 17.53 | 39.65 | 35.57 | 433.58 |
| NEW40 | CGTTGTAGTATAGTGGTAAGTAT | 24 | 49.10 | -31.10 | 7.38 | 17.88 | 13.45 | 111.45 |
| NEW41 | GCTTTCTCTTCTTCTGTC | 18 | 47.70 | -43.70 | 0 | 37.32 | 21.69 | 11.39 |

Table S5 Data summary of two degradome sequencing from litchi pericarp

|  | | | S1S4 | | | S2S3 | |
| --- | --- | --- | --- | --- | --- | --- | --- |
|  |  |  | Total | % of Total | Total | | % of Total |
| Raw Reads | | 8127257 | | / | 7112553 | | / |
| reads < 15nt after removing 3 adaptor | 50035 | | | 0.62% | 47785 | | 0.67% |
| Mappable Reads | 8077222 | | | 99.38% | 7064768 | | 99.33% |
| Unique Raw Reads | 3551258 | | | / | 3818329 | | / |
| Unique reads < 15nt after removing 3 adaptor | 27045 | | | 0.76% | 29525 | | 0.77% |
| Unique Mappable Reads | 3524213 | | | 99.24% | 3788804 | | 99.23% |
| Transcript Mapped Reads | 3788036 | | | 46.61% | 2844214 | | 39.99% |
| Unique Transcript Mapped Reads | 1525517 | | | 42.96% | 1404529 | | 36.78% |
| Number of input Transcript | 51089 | | | / | 51089 | | / |
| Number of Covered Transcript | 27455 | | | 53.74% | 25287 | | 49.50% |

| Table S6 miRNA targets identified from the degradome sequencing | | | | | | |
| --- | --- | --- | --- | --- | --- | --- |
| **SmallRNA** | **Target** | **Alignment Score** | **Cleavage Site** | **S1S4** | **S2S3** | **target gene family** |
|  |  |  |  | **Category** | **Category** |  |
| lch-miR156 | Unigene0018408 | 1.5 | 455 | 0 |  | Squamosa promoter-binding protein |
|  | Unigene0018982 | 2 | 726 | 0 | 2 | Squamosa promoter-binding protein |
|  | Unigene0049464 | 4 | 275 | 0 | 1 | Plastid-specific 30S ribosomal protein 1 |
|  | Unigene0026828 | 4 | 87 | 4 |  | E3 ubiquitin-protein ligase RNF167-like |
|  | Unigene0028336 | 4 | 214 | 0 | 2 | phosphoenolpyruvate carboxylase |
|  | Unigene0018577 | 3.5 | 97 | 4 |  | glycerophosphodiester phosphodiesterase GDE1 |
|  | Unigene0034516 | 3 | 223 | 2 |  | stem-specific protein TSJT1-like |
| lch-miR159 | Unigene0005792 | 4 | 259 | 4 |  | UDP-glucose flavonoid glucosyl-transferase |
|  | Unigene0002687 | 4 | 474 | 4 |  | DNA mismatch repair protein Msh6-1-like |
|  | Unigene0017650 | 2 | 662 |  | 0 | transcription factor GAMYB |
|  | Unigene0025140 | 3 | 1417 | 4 | 4 | transcription factor TCP4-like |
| lch-miR160 | Unigene0025834 | 1 | 1466 |  | 0 | auxin response factor 17-like |
|  | Unigene0017016 | 1 | 380 |  | 4 | auxin response factor 18-like |
| lch-miR164 | Unigene0016839 | 4 | 424 | 4 |  | vesicle-associated membrane protein 714 |
|  | Unigene0019191 | 4 | 162 | 4 |  | high-affinity nitrate transporter 3.1-like |
|  | Unigene0029429 | 4 | 162 |  | 4 | sorting nexin-2-like |
| lch-miR166 | Unigene0040623 | 3 | 154 | 4 |  | peroxidase N |
|  | Unigene0030215 | 2.5 | 984 |  | 0 | homeobox-leucine zipper protein HOX32-like isoform 3 |
|  | Unigene0029197 | 2.5 | 1935 |  | 4 | homeobox-leucine zipper protein ATHB-15-like isoform 1 |
|  | Unigene0029159 | 3 | 29 |  | 4 | homeobox-leucine zipper protein REVOLUTA |
| lch-miR168 | Unigene0029791 | 4 | 723 | 4 |  | transportin-3 |
|  | Unigene0047074 | 4 | 1149 | 4 |  | transportin-3 |
|  | Unigene0022120 | 4 | 3024 |  | 4 | nbs-lrr resistance protein |
|  | Unigene0022117 | 4 | 2952 |  | 4 | nbs-lrr resistance protein |
|  | Unigene0022119 | 4 | 2904 |  | 4 | nbs-lrr resistance protein |
| lch-miR169 | Unigene0020696 | 3.5 | 810 | 2 |  | probable mitochondrial chaperone BCS1-B-like |
|  | Unigene0030578 | 2.5 | 1063 | 2 |  | protein disulfide-isomerase |
|  | Unigene0021878 | 4 | 204 | 4 |  | nitric oxide synthase-interacting protein-like |
|  | Unigene0049178 | 4 | 870 | 2 |  | F-box protein PP2-B1 |
|  | Unigene0027192 | 4 | 1225 | 4 | 4 | probable receptor-like protein kinase At5g15080-like |
|  | Unigene0028002 | 3.5 | 1312 |  | 2 | nuclear transcription factor Y subunit A-1-like |
|  | Unigene0049411 | 3 | 382 |  | 4 | auxin-induced protein 22D |
| lch-miR171 | Unigene0030534 | 1 | 1590 | 0 |  | GRAS family transcription factor |
|  | Unigene0022328 | 1 | 467 | 2 | 0 | GRAS family protein, partial |
|  | Unigene0007515 | 4 | 53 | 2 |  | auxin response factor |
|  | Unigene0030531 | 1 | 872 | 4 |  | SCL6 |
|  | Unigene0030532 | 1.5 | 1774 | 4 |  | SCL6 |
|  | Unigene0022332 | 3 | 321 |  | 4 | ADP-ribosylation factor-related protein 1-like |
| lch-miR172 | Unigene0016486 | 3 | 797 | 2 | 4 | proteasome subunit alpha type-2-A-like |
|  | Unigene0049536 | 4 | 1433 | 4 | 4 | glucose-6-phosphate dehydrogenase 6 |
|  | Unigene0023087 | 3 | 1141 |  | 4 | ethylene responsive factor, partial |
|  | Unigene0018778 | 4 | 321 |  | 4 | Protein kinase |
|  | Unigene0049277 | 4 | 2902 |  | 4 | P-loop containing nucleoside triphosphate hydrolase domain-containing protein |
|  | Unigene0027584 | 1 | 1570 |  | 4 | ethylene-responsive transcription factor RAP2-7-like |
|  | Unigene0027583 | 1.5 | 1615 |  | 4 | ethylene-responsive transcription factor RAP2-7-like |
| lch-miR319 | Unigene0049868 | 4 | 1212 | 2 |  | RNase H domain-containing protein |
|  | Unigene0029268 | 4 | 1183 | 2 |  | RNase H domain-containing protein |
|  | Unigene0029475 | 3.5 | 1562 | 4 |  | PREDICTED: U3 small nucleolar RNA-associated protein 15 |
|  | Unigene0025140 | 3.5 | 1418 | 4 |  | PREDICTED: transcription factor TCP4-like |
|  | Unigene0025141 | 3.5 | 1418 | 4 |  | PREDICTED: transcription factor TCP4-like |
|  | Unigene0017650 | 3 | 663 |  | 0 | PREDICTED: transcription factor GAMYB [Vitis vinifera] |
|  | Unigene0027380 | 1.5 | 618 |  | 4 | PREDICTED: indole-3-glycerol phosphate synthase, chloroplastic |
|  | Unigene0021579 | 4 | 678 |  | 4 | Ankyrin repeat domain-containing protein |
| lch-miR390 | Unigene0024765 | 4 | 259 | 4 | 4 | PREDICTED: acyl-coenzyme A oxidase 3, peroxisomal |
|  | Unigene0029327 | 4 | 511 |  | 4 | PREDICTED: 70 kDa peptidyl-prolyl isomerase |
|  | Unigene0033663 | 3.5 | 1407 |  | 4 | PREDICTED: ribosome biogenesis protein BMS1 homolog |
| lch-miR393 | Unigene0049137 | 4 | 716 | 4 |  | PREDICTED: serine hydroxymethyltransferase, mitochondrial |
|  | Unigene0035106 | 3.5 | 1230 | 4 |  | PREDICTED: [UDP-forming] 11-like |
|  | Unigene0019006 | 1 | 11 |  | 0 | transport inhibitor response 1 [Dimocarpus longan] |
|  | Unigene0051000 | 4 | 3817 |  | 2 | TMV-associated RING finger protein [Nicotiana tabacum] |
| lch-miR395 | Unigene0022970 | 4 | 679 |  | 4 | PREDICTED: FK506-binding protein 59 isoform 1 |
| lch-miR396 | Unigene0032731 | 4 | 2354 | 4 |  | nbs-lrr resistance protein |
|  | Unigene0027864 | 4 | 1201 | 2 | 4 | PREDICTED: serine/threonine-protein kinase SRK2B isoform 1 |
|  | Unigene0044715 | 4 | 14 | 4 |  | - |
|  | Unigene0015492 | 4 | 922 | 4 |  | PREDICTED: elongation factor G, chloroplastic-like |
|  | Unigene0029935 | 4 | 583 | 4 |  | PREDICTED: l-Ala-D/L-Glu epimerase |
|  | Unigene0027262 | 4 | 328 | 2 |  | JHS03A10.2 [Jatropha curcas] |
|  | Unigene0037844 | 3.5 | 170 | 2 |  | - |
|  | Unigene0006054 | 3 | 43 |  | 0 | PREDICTED: uncharacterized protein LOC100787567 [Glycine max] |
|  | Unigene0046871 | 4 | 508 | 4 |  | PREDICTED: flavanone 3-dioxygenase-like [Glycine max] |
|  | Unigene0025264 | 3.5 | 772 | 4 | 4 | RNAse E/G-like protein [Arabidopsis thaliana] |
|  | Unigene0025258 | 4 | 407 | 3 | 3 | contains similarity to Solanum lycopersicum (tomato) wound-induced protein |
|  | Unigene0025257 | 4 | 260 | 3 | 2 | contains similarity to Solanum lycopersicum (tomato) wound-induced protein |
|  | Unigene0048627 | 4 | 407 | 3 | 2 | contains similarity to Solanum lycopersicum (tomato) wound-induced protein |
|  | Unigene0047124 | 3.5 | 1007 |  | 2 | PREDICTED: UPF0082 protein At2g25830 [Vitis vinifera] |
|  | Unigene0031711 | 4 | 254 |  | 2 | nuclear matrix constituent protein 1 [Daucus carota] |
|  | Unigene0032138 | 3 | 1251 |  | 4 | serine/threonine protein phosphatase |
| lch-miR397 | Unigene0042679 | 4 | 460 | 4 |  | tRNA ligase [Solanum melongena] |
| lch-miR398 | Unigene0021129 | 4 | 1165 |  | 4 | Chaperone protein dnaJ [Medicago truncatula] |
| lch-miR399 | Unigene0028509 | 4 | 256 | 2 |  | PREDICTED: patellin-3-like [Vitis vinifera] |
|  | Unigene0048628 | 3.5 | 461 | 4 |  | Mandelonitrile lyase [Medicago truncatula] |
|  | Unigene0022367 | 2.5 | 50 |  | 2 | PREDICTED: probable ubiquitin-conjugating enzyme E2 24-like |
| lch-miR408 | Unigene0003383 | 4 | 61 | 4 |  | conserved hypothetical protein |
|  | Unigene0030037 | 4 | 1342 | 2 | 4 | fasciclin-like arabinogalactan protein 19 |
|  | Unigene0020875 | 3.5 | 1089 | 2 |  | PREDICTED: transmembrane protein 56-B [Vitis vinifera] |
| lch-miR477 | Unigene0026202 | 4 | 333 | 4 |  | PREDICTED: probable phosphatidylinositol 4-kinase type 2-beta At1g26270 isoform 1 |
|  | Unigene0029510 | 3 | 479 |  | 4 | PREDICTED: U-box domain-containing protein 34-like |
|  | Unigene0029677 | 4 | 629 | 4 | 2 | PREDICTED: auxilin-related protein 2-like |
|  | Unigene0029678 | 4 | 599 | 4 | 2 | PREDICTED: auxilin-related protein 2-like |
| lch-miR827 | Unigene0025365 | 4 | 1090 | 2 |  | PREDICTED: aspartate aminotransferase, mitochondrial |
| lch-miR828 | Unigene0039117 | 1 | 97 | 4 | 0 | MYB |
| lch-miR3630 | Unigene0049773 | 4 | 1100 | 2 |  | PREDICTED: ankyrin repeat domain-containing protein 13C |
|  | Unigene0028104 | 4 | 717 | 4 |  | PREDICTED: probable ADP-ribosylation factor |
|  | Unigene0031905 | 4 | 404 | 4 |  | AGAMOUS-like protein [Mangifera indica] |
|  | Unigene0027827 | 3 | 1839 | 4 |  | PREDICTED: kinesin-like protein KIF2A-like |
|  | Unigene0027804 | 4 | 236 | 4 |  | PREDICTED: probable xyloglucan endotransglucosylase |
|  | Unigene0029932 | 4 | 196 | 4 |  | PREDICTED: U-box domain-containing protein 4-like |
|  | Unigene0050001 | 4 | 441 | 4 |  | protein transport protein SFT1 |
|  | Unigene0022423 | 4 | 588 | 4 | 4 | 2-dehydro-3-deoxyphosphoheptonate aldolase |
|  | Unigene0004802 | 4 | 25 |  | 4 | SYNC1 protein |
|  | Unigene0033707 | 4 | 2731 |  | 4 | kinase family protein |
|  | Unigene0025742 | 4 | 1209 |  | 4 | PREDICTED: protein NSP-INTERACTING KINASE 1 |
|  | Unigene0029701 | 4 | 228 |  | 4 | PREDICTED: auxin response factor 9-like |
| lch-miR482 | Unigene0047942 | 4 | 239 | 2 | 4 | - |
|  | Unigene0008558 | 4 | 90 |  | 0 | kinase, putative |
|  | Unigene0038759 | 3 | 51 |  | 0 | - |
|  | Unigene0004717 | 4 | 86 | 4 | 0 | - |
|  | Unigene0026537 | 4 | 507 | 4 | 4 | PREDICTED: probable serine/threonine-protein kinase Cx32 |
|  | Unigene0050046 | 4 | 2554 | 4 | 4 | RING finger protein |
|  | Unigene0050642 | 3 | 237 |  | 1 | PREDICTED: cysteine-rich receptor-like protein |
|  | Unigene0048636 | 4 | 802 |  | 1 | nbs-lrr resistance protein |
|  | Unigene0046253 | 3 | 130 |  | 4 | - |
|  | Unigene0000083 | 2.5 | 116 |  | 4 | cc-nbs-lrr resistance protein |
| lch-miR1448 | Unigene0047942 | 4 | 240 | 0 |  | - |
|  | Unigene0004717 | 3 | 86 | 4 | 0 | - |
|  | Unigene0008558 | 3 | 90 |  | 0 | kinase, putative |
|  | Unigene0038759 | 4 | 51 |  | 0 | - |
|  | Unigene0047942 | 4 | 239 | 2 | 4 | - |
|  | Unigene0050642 | 4 | 240 | 0 |  | PREDICTED: cysteine-rich receptor-like protein kinase 10-like |
|  | Unigene0051016 | 4 | 240 | 0 |  | PREDICTED: cysteine-rich receptor-like protein kinase 10-like |
|  | Unigene0046037 | 2 | 12 |  | 0 | - |
|  | Unigene0046253 | 1 | 131 | 2 |  | - |
|  | Unigene0017362 | 4 | 286 | 4 |  | PREDICTED: ATP-dependent DNA helicase recG-like |
|  | Unigene0017363 | 4 | 286 | 4 |  | PREDICTED: ATP-dependent DNA helicase recG-like |
| PC-5p-1920_1330 | Unigene0012421 | 4 | 371 | 2 |  | PREDICTED: probable calcium-binding protein CML22 |
| PC-3p-1800_1402 | Unigene0022988 | 3.5 | 1688 | 2 |  | PREDICTED: calcium-dependent protein kinase 16 |
| PC-3p-341_5690 | Unigene0024256 | 3.5 | 869 |  | 0 | BSD domain containing protein |
| PC-5p-32_58562 | Unigene0001591 | 3 | 220 |  | 4 | tir-nbs-lrr resistance protein |
| PC-5p-100_18950 | Unigene0018105 | 4 | 189 |  | 4 | PREDICTED: transcription domain-associated protein |
| mdm-MIR156u-p3_1ss20TG | Unigene0012688 | 2.5 | 1153 | 4 | 2 | nuclear RNA binding protein |
|  | Unigene0011913 | 4 | 791 | 3 | 3 | allergenic-related protein [Manihot esculenta] |
|  | Unigene0018461 | 4 | 635 | 2 | 1 | CHI |
|  | Unigene0006672 | 4 | 226 |  | 2 | PREDICTED: uncharacterized protein |
| mtr-MIR2592bj-p3_1ss12TC | Unigene0027874 | 4 | 71 |  | 4 | PREDICTED: thioredoxin-like protein HCF164 |
|  | Unigene0024960 | 4 | 2595 |  | 4 | PREDICTED: probable serine/threonine-protein kinase DDB |
|  | Unigene0024961 | 4 | 1651 |  | 4 | PREDICTED: probable serine/threonine-protein kinase DDB |
|  | Unigene0023299 | 4 | 817 | 2 | 2 | f-box family protein |
|  | Unigene0048370 | 4 | 1693 | 4 |  | PREDICTED: choline transporter-like protein 3 |
|  | Unigene0016037 | 4 | 772 | 2 | 4 | PREDICTED: 31 kDa ribonucleoprotein |
| mes-MIR171b-p3 | Unigene0022328 | 1 | 467 | 2 | 0 | GRAS family protein, partial |
|  | Unigene0030534 | 1 | 1590 | 0 |  | GRAS family transcription factor |
|  | Unigene0022328 | 1 | 467 | 2 | 0 | GRAS family protein, partial |
|  | Unigene0030531 | 1 | 872 | 4 |  | SCL6 [Citrus trifoliata] |
|  | Unigene0030532 | 1 | 1774 | 4 |  | SCL6 [Citrus trifoliata] |
| PC-5p-1920_1330 | Unigene0012421 | 4 | 371 | 2 |  | PREDICTED: probable calcium-binding protein CML22 |
| PC-3p-1800_1402 | Unigene0022988 | 3.5 | 1688 | 2 |  | PREDICTED: calcium-dependent protein kinase 16 |
| PC-3p-341_5690 | Unigene0024256 | 3.5 | 869 |  | 0 | BSD domain containing protein |
| PC-5p-32_58562 | Unigene0001591 | 3 | 220 |  | 4 | tir-nbs-lrr resistance protein |
| PC-5p-100_18950 | Unigene0018105 | 4 | 189 |  | 4 | PREDICTED: transformation/transcription domain-associated protein |
| mdm-MIR156u-p3_1ss20TG | Unigene0012688 | 2.5 | 1153 | 4 | 2 | nuclear RNA binding protein [Solanum tuberosum] |
|  | Unigene0011913 | 4 | 791 | 3 | 3 | allergenic-related protein [Manihot esculenta] |
|  | Unigene0018461 | 4 | 635 | 2 | 1 | CHI |
|  | Unigene0006672 | 4 | 226 |  | 2 | PREDICTED: uncharacterized protein |
| mtr-MIR2592bj-p3_1ss12TC | Unigene0027874 | 4 | 71 |  | 4 | PREDICTED: thioredoxin-like protein HCF164 |
|  | Unigene0024960 | 4 | 2595 |  | 4 | PREDICTED: probable serine |
|  | Unigene0024961 | 4 | 1651 |  | 4 | PREDICTED: probable serine |
|  | Unigene0023299 | 4 | 817 | 2 | 2 | f-box family protein trichocarpa] |
|  | Unigene0048370 | 4 | 1693 | 4 |  | PREDICTED: choline transporter-like protein 3 |
|  | Unigene0016037 | 4 | 772 | 2 | 4 | PREDICTED: 31 kDa ribonucleoprotein, chloroplastic isoform 1 |
| mes-MIR171b-p3 | Unigene0022328 | 1 | 467 | 2 | 0 | GRAS family protein, partial [Dimocarpus longan] |
|  | Unigene0030534 | 1 | 1590 | 0 |  | GRAS family transcription factortranscription factor |
|  | Unigene0022328 | 1 | 467 | 2 | 0 | GRAS family protein, partial |
|  | Unigene0030531 | 1 | 872 | 4 |  | SCL6 [Citrus trifoliata] |
|  | Unigene0030532 | 1 | 1774 | 4 |  | SCL6 [Citrus trifoliata] |
